# Human Contacts with Oral Rabies Vaccine Baits Distributed for Wildlife Rabies Management — Ohio, 2012

**Published:** 2013-04-12

**Authors:** Frank Kellogg, Nancy Niehus, Mary DiOrio, Kathleen Smith, Richard Chipman, Jordona Kirby, Jesse Blanton, Jessie Dyer, Richard Franka, Kim Hummel, Sergio Recuenco, Charles Rupprecht, Ryan Wallace, Neil M. Vora

**Affiliations:** Lake County General Health District; Ohio Dept of Health; Wildlife Svcs, US Dept of Agriculture; Div of High-Consequence Pathogens and Pathology, National Center for Emerging and Zoonotic Infectious Diseases; EIS officers, CDC

Baits laden with oral rabies vaccines are important for the management of wildlife rabies in the United States (1). In August 2012, the Wildlife Services program of the U.S. Department of Agriculture’s Animal and Plant Health Inspection Service began a field trial involving limited distribution of a new oral rabies vaccine bait in five states, including Ohio. The vaccine consisted of live recombinant human adenovirus type 5 vector, expressing rabies virus glycoprotein (AdRG1.3) (Onrab). A previously used oral rabies vaccine consisting of a live recombinant vaccinia vector, expressing rabies virus glycoprotein (V-RG) (Raboral V-RG) (2,3), was distributed in other areas of Ohio. To monitor human contacts and potential vaccine virus exposure, surveillance was conducted by the Ohio Department of Health, local Ohio health agencies, and CDC. During August 23–September 7, 2012, a total of 776,921 baits were distributed in Ohio over 4,379 square miles (11,341 square kilometers). During August 24–September 12, a total of 89 baits were reported found by the general public, with 55 human contacts with baits identified (some contacts involved more than one bait). In 27 of the 55 human contacts, the bait was not intact, and a barrier (e.g., gloves) had not been used to handle the bait, leaving persons at risk for vaccine exposure and vaccine virus infection. However, no adverse events were reported. Continued surveillance of human contacts with oral rabies vaccine baits and public warnings to avoid contact with baits are needed because of the potential for vaccine virus infection.

Wildlife accounts for more than 90% of the rabid animals reported in the United States, and raccoons are the species most frequently reported (4). Oral rabies vaccination is an effective strategy to prevent the spread of rabies in reservoirs such as raccoons, coyotes, and foxes. Baits laden with oral rabies vaccine are distributed in strategic areas where target species can find and consume the baits, thereby releasing vaccine into their oral cavity. Oral rabies vaccination has contributed to the elimination of the red fox rabies virus variant and the canine rabies virus variant from several European countries and the United States, respectively, and has helped to prevent any appreciable spread of the raccoon rabies virus variant in the eastern United States (1). V-RG has been used in the United States since 1990, with approximately 138 million doses released to date. Baiting strategies have attempted to minimize human contact with V-RG baits because of the risk for infection with the V-RG vaccine virus; only two human vaccinia infections have been reported from V-RG exposure (3,5,6). AdRG1.3 is an alternative to V-RG that might have a different human safety profile given the high prevalence of antibodies in humans to human adenovirus type 5 and the mild illness that typically results from infection with this virus (7). AdRG1.3 has been integrated successfully into raccoon rabies management programs in Canada and has shown promise when used at higher bait densities for eliminating residual rabies foci in skunks (8,9).

Before and during the 2012 distribution of baits, the Ohio Department of Health, Wildlife Services, and Ohio local health jurisdictions used print media, television, radio, and the Internet to raise awareness and provide guidance to the public regarding what to do if a bait was found by a person or domestic animal. Despite these efforts, 75% of persons who came in contact with a bait were unaware of the baiting operation. A human contact was recorded when a person reported either seeing or coming into physical contact with a single bait or multiple baits with or without a barrier such as gloves. Contacts were reported by calling the toll-free telephone numbers printed on all baits or by contacting local health departments directly.

Persons who came into physical contact with an intact bait (i.e., a bait that was neither punctured nor leaking) did not require further follow-up, even if they did not use a barrier such as gloves, because vaccine exposure was not likely to have occurred. However, persons who came into physical contact with a bait that was not intact and who did not use a barrier such as gloves were considered to be potentially exposed to vaccine and at risk for vaccine virus infection. Attempts were made to contact all persons potentially exposed to vaccine 21 days after the event to ensure that their symptoms, if any, were reported. Persons who were immunocompromised, pregnant, aged <12 years, or cognitively impaired and persons with dermatologic conditions or a history of vaccine exposure to a mucosal membrane were contacted sooner than 21 days after the potential exposure.

During August 23–September 7, 2012, a total of 776,921 baits (272,034 AdRG1.3 and 504,887 V-RG baits) (Figure) were distributed by automobile in urban areas and by aircraft in rural areas of Ohio over an area of 4,379 square miles (11,341 square kilometers). A total of 89 baits were reported found by the general population during August 24–September 12 (11.5 baits found per 100,000 baits distributed). Fifteen of the baits found were AdRG1.3 (5.5 per 100,000 AdRG1.3 baits distributed), and 74 were V-RG (14.7 per 100,000 V-RG baits distributed) (p<0.001).

Among the 89 baits found, 55 human contacts occurred (some human contacts involved more than one bait). Fourteen of the human contacts were with AdRG1.3 baits, and 41 were with V-RG baits. Among the 55 human contacts, 27 involved potential vaccine exposures. Among the AdRG1.3 bait contacts, 79% resulted in potential vaccine exposure, compared with 39% of V-RG bait contacts (odds ratio: 5.7; 95% confidence interval: 1.4–23.8) (Table 1). Only 5.8% of persons physically contacting a bait used a barrier such as gloves.

Fifty-four of the human contacts were reported through 47 telephone calls on the toll-free numbers (more than one human contact was reported on some calls). An additional human contact was reported directly to a local health department. The total report rate was 6.2 reports per 100,000 baits distributed, with 4.4 reports per 100,000 AdRG1.3 baits distributed and 7.1 reports per 100,000 V-RG baits distributed (Table 2).

Five of the persons who had potential vaccine exposures also had one of the conditions that required closer follow-up. Three of these incidents occurred with AdRG1.3 and involved a boy aged 11 years, a pregnant woman, and a woman with eczema. The other two incidents occurred with V-RG in women who had autoimmune conditions and were on immunosuppressive medications. No adverse events were reported among these five persons or among the other persons who contacted baits.

A total of 38 (79%) of the 48 reports of human contact involved domestic animals, and all of the animals were dogs. One animal adverse event resulted from an AdRG1.3 bait temporarily obstructing a dog’s airway, but the dog survived. Two other adverse events were reported for V-RG baits in which the dogs regurgitated the baits.

## Editorial Note

Surveillance during rabies vaccine baiting operations in Ohio suggests that human and domestic animal contacts with baits are rare. In 2010 and 2011, totals of 774,714 and 863,215 baits were distributed in Ohio, respectively, compared with 776,921 in 2012 (10). Overall, fewer human contacts with baits were reported in 2012 than in the preceding 2 years: 55 in 2012, compared with 83 in both 2010 and 2011 (Ohio Department of Health, unpublished data, 2012).

Multistate surveillance of contacts with V-RG baits during 2001–2009 revealed 6.9 V-RG baits found per 100,000 V-RG baits distributed for the study period, compared with 14.7 V-RG baits found per 100,000 V-RG baits distributed in Ohio in 2012. This same multistate surveillance system found 3.5 reports of V-RG bait contacts per 100,000 V-RG baits distributed during 2001–2009 (3), compared with 7.1 reports per 100,000 V-RG baits distributed in Ohio in 2012. Similar report rates have been observed previously in other states (3).

In 2012, AdRG1.3 was distributed for the first time in Ohio. The rate of 4.4 reports of AdRG1.3 bait contacts per 100,000 baits distributed was higher than rates observed in Canada (8,9) and in the first AdRG1.3 field trial in the United States in rural West Virginia in 2011 (Wildlife Services, U.S. Department of Agriculture, unpublished data, 2012). However, no adverse events were reported as a result of human contacts with baits in Ohio, Canada, or West Virginia (Wildlife Services, U.S. Department of Agriculture, unpublished data, 2013) (8,9). Because the risk for infection arises from exposure to vaccine virus rather than from contact with an intact bait, the higher proportion of human contacts that resulted in potential vaccine exposure with AdRG1.3 baits compared with V-RG baits deserves further evaluation.

The low percentage of persons who were aware of the baiting operation at the time of bait contact suggests that public outreach strategies should be evaluated and modified to enhance public awareness. Similar low rates of awareness about baiting operations have been reported in the past (3). In addition, only 5.8% of persons physically contacting a bait reported using a barrier such as gloves to handle baits, underscoring the need to raise awareness about the potential risk of handling baits without protection.

What is already known on this topic?

Baits laden with oral rabies vaccine play an important role in the management of rabies in wildlife. An oral rabies vaccine consisting of a recombinant vaccinia vector (V-RG) has been used in the United States for over 20 years; during this time only two cases of human vaccinia infection from human contact with vaccine in the baits have been reported. An oral rabies vaccine consisting of a recombinant human adenovirus type 5 vector (AdRG1.3) is now being field tested in the United States to assess its safety and immunogenicity.

What is added by this report?

This is the first published report of human contacts with AdRG1.3 baits in the United States. In 2012, a total 272,034 AdRG1.3 and 504,887 V-RG baits were distributed in Ohio. A total of 55 human contacts with the baits were reported, with potential vaccine exposure in 27 of the human contacts (11 with AdRG1.3 and 16 with V-RG). No adverse events were reported.

What are the implications for public health practice?

Ongoing surveillance is needed of human contacts with AdRG1.3 and V-RG baits. The low level of awareness about baiting operations among those who came into contact with baits suggests a need for improved public outreach before distributing baits.

## Figures and Tables

**FIGURE:**
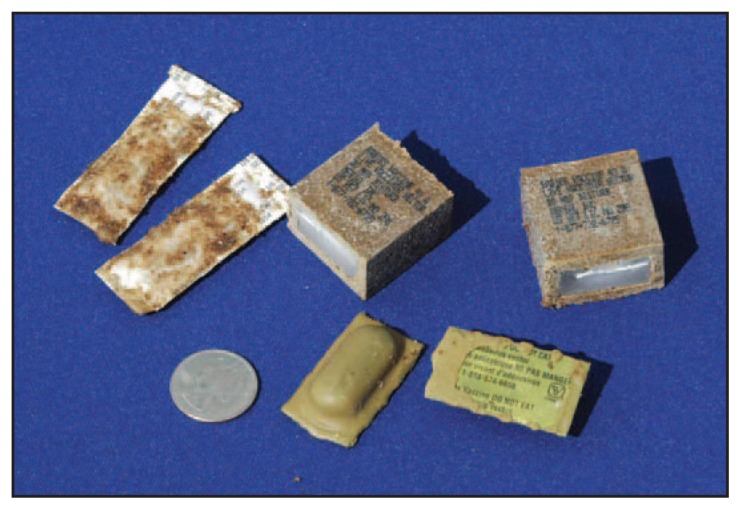
Types of oral rabies vaccine baits* distributed by Wildlife Services of the U.S. Department of Agriculture’s Animal and Plant Health Inspection Service — Ohio, 2012 Photo/U.S. Department of Agriculture, Animal and Plant Health Inspection Service, Wildlife Services * Two types of oral rabies vaccines were distributed in different areas of Ohio: a new oral rabies vaccine (AdRG1.3) and one that has been in use since 1990 (V-RG). Clockwise from upper left: two V-RG coated sachets, two V-RG fishmeal polymer blocks, two AdRG1.3 polyvinyl chloride blister packs. A U.S. quarter is shown to illustrate the size of the baits.

**TABLE 1:** Reported number of human contacts with oral rabies vaccine baits and number and percentage of contacts with potential vaccine exposure, by year and bait type — Ohio, 2010–2012

Year/Bait type	No. of human contacts	No. of contacts with potential vaccine exposure	(%)
2010*	83	37	(45)
2011*	83	29	(35)
2012 (total)	55	27	(49)
AdRG1.3	14	11	(79)
V-RG	41	16	(39)

**Abbreviations:** AdRG1.3 = human adenovirus type 5-rabies glycoprotein recombinant vaccine; V-RG = vaccinia-rabies glycoprotein recombinant vaccine.

* During 2010 and 2011, only V-RG was distributed.

**TABLE 2:** Reported number of oral rabies vaccine baits distributed and later found and numbers of human contacts and reports received, by bait type — Ohio, 2012

Bait type	No. of baits distributed	No. of baits found	No. of human contacts reported	No. of reports received	Reports received per 100,000 baits
AdRG1.3	272,034	15	14	12	4.4
V-RG	504,887	74	41	36	7.1

**Abbreviations:** AdRG1.3 = human adenovirus type 5-rabies glycoprotein recombinant vaccine; V-RG = vaccinia-rabies glycoprotein recombinant vaccine.
